# Supplementary material for: Determinants predicting the electronic medical record adoption in healthcare: A SEM-Artificial Neural Network approach
Source: PLoS One. 2022 Aug 16;17(8):e0272735. doi: 10.1371/journal.pone.0272735 (PMC9380954; doi:10.1371/journal.pone.0272735)
Supplement: Attachment — Submitted filename: Response to reviewers.docx [file pone.0272735.s001.docx]

**Original Manuscript ID:** PONE-D-22-05312

**Original Article Title:** “Determinants Predicting the Electronic Medical Record Adoption in Healthcare: A SEM- Artificial Neural Network Approach”

**To:** PLOS ONE Editor

**Re:** Response to reviewers

Dear Editor,

The authors are really very grateful to the Journal Editor efforts in essentially discussing the paper reviews with the reviewers and identifying the further significant points for enhancements. The authors would also like to sincerely thank the reviewers for their valuable remarks and careful feedback which helped them to significantly enhance this work and its presentation. Regardless of the final outcome, the authors sincerely thank the editor and reviewers for supporting them. The productive and valuable remarks enable them to update many parts of the paper as shown by the responses to each comment. Besides, all the updated parts in the manuscript were highlighted in yellow color in order to be easily tracked by the editor and reviewers.

Thank you again for allowing a resubmission of our manuscript, with an opportunity to address the reviewers’ comments.

We are uploading (a) our point-by-point response to the comments (below) (response to reviewers), (b) an updated manuscript with yellow highlighting indicating changes (*Revised Manuscript with Track Changes*), and (c) a clean updated manuscript without highlights (*Manuscript).*

Best regards,

Almarzouqi et al.

| **Authors’ Response to Reviewer 1:**  The authors are really very grateful to the feedback and comments raised by the reviewer which really assist them to significantly enhance this work and its presentation. The productive and valuable remarks enable us to update many parts of the paper as shown by the responses to each comment. Our responses are mentioned below under each comment raised by the reviewer and it is written in (Times New Roman font, Italic, blue color). Besides, all the updated parts in the manuscript were highlighted in yellow color in order to be easily tracked by the reviewers. | | |
| --- | --- | --- |
| **#** | **Reviewers’ comments** | **Authors’ response** |
| **1** | The last two sentences in the “Abstract” seem ‘not necessary’. The first one says, “In addition, the study offers methodological significance as it proves the efficiency of the ANN algorithm in determining non-linear linkages”, such a conclusion is a bit beyond the scope of the paper; after all, this is not a ‘pure’ machine learning study. As for the second one “It can also be concluded that the adoption of an EMR system is in high demand in the healthcare setting as it enhances the transmission and sharing of information within and between healthcare institutions.”, I would say this is a ‘trivial’ conclusion. | *The authors are really very grateful to the reviewer for notifying them about these issues. In the revised version of the manuscript, the last two sentences have been deleted in the abstract section.* |
| **2** | Is “Literature Review” mentioned in line 117 a convenient title to the successive paragraphs? I am not able to see the ‘border’ between the section entitled “Introduction” and the other one entitled “Literature Review”. I would remove that title and keep them all under “Introduction”. In the same context, lines 118-125 contain nothing, but a redundancy of information already discussed in the “Introduction”; I would remove them as well. | *In the revised version of the paper, the introduction section has been merged with the literature review section. On the recommendation of the reviewer, the redundancy of information in lines 118-125 has been removed.* |
| **3** | • Confusion between developing and developed countries:  What is surprising in this manuscript is the implicit mention that UAE is a developing country!!! For example, it is mentioned in Line 592 “with more significance in the UAE and other developing countries” ... I ask the authors to clarify this point, I would definitely disagree to classify UAE as a developing country, it is one of the most developed countries in the world. In line 79, after talking about developing countries, authors suddenly moved to discuss the use of EMR in the USA! The same occurs in some other instances in the manuscript. Incentives, challenges, effects… of adopting EMR in developed countries should be discussed separately from those in developing countries. Well, I understand that many factors could be common, but that confusion should be addressed. In other words, authors are asked to explain explicitly and plainly the problem of the EMR overall (common factors amongst all countries), then to differentiate between the scope of the problem in developing and developed countries and clarify the case of UAE.  Have the questionnaires used in this paper been used somewhere else? I mean in other particle? | *Since its Federation in 1971, the UAE has* ***developed rapidly*** *and is now noted for its modern infrastructure, international events and status as a trade and transport hub.* *The UAE ranked first in the Arab world and 15th globally in terms of its ability to attract foreign direct investment, according to the results of the Global Foreign Direct Investment Report for the year 2021 issued by the United Nations Conference on Trade and Development.* *Therefore, I agree with the reviewer’s opinion in the classification of the Emirates among the developed countries.*  *The case has been corrected.* |
| **4** | Acronyms: Some abbreviations are used – some of them too many times - without explanation in their first instances: UTAUT is never explained as Unified Theory of Acceptance and Use of Technology. Speaking of which, I was wondering if the terms mentioned between brackets in line 173 as “(user behaviour and acceptance analysis)” next to UTAUT meant to be a description of some the model’s characteristics. I believe it is confusing a bit. TAM is never explained as Technology Acceptance Model. ANN is explained as Artificial Neural Networks in line 339, not in the first instance it was used. SEM is never explained as Structural Equation Modeling, ICT is never explained as Information and Communications Technology NHIN is never explained as Nationwide Health Information Network PLS-SEM is never explained as Partial Least Squares Structural Equation Modeling IPMA is explained as Importance-Performance Map Analysis in line 529, not in the first instance it was used. And BI… Please make sure you pass through all acronyms and double-check. | *The authors are really very grateful to the reviewer for notifying them about these issues. In the revised version of the manuscript, all acronyms have been checked.* |
| **5** | Minor mistakes/comments: In line 86, I think there is something missing in the English… “there is research handling” In line 94, what research in “the research”? the current one? If not, what reference? One expects a reference in line 113, after “technology”. Same applies to line 115 after “study” and line 140 after “data”. In line 173, “The researcher” is not well fitted in the sentence. In lines 178-179: I think capital letters are not necessary here. In line 190, a comma is to be inserted after (37). In line 225, period to be removed after “connection”. In table 1, I think “frequency” is mentioned twice as a column header wrongly. In Table 5, “Mod” to be written as “Model” In line 545, who is “the researcher”? same question in line 637. In line 552, I suppose something is wrong “Aligning with this is (37) study proves” In line 607, I suppose there is a missing word after “main”. | *The authors are really very grateful to the reviewer for notifying them about these issues. In the revised version of the manuscript, all mistakes have been fixed. .* |

| **Authors’ Response to Reviewer 2:**  The authors are really very grateful to the feedback and comments raised by the reviewer which really assist them to significantly enhance this work and its presentation. The productive and valuable remarks enable us to update many parts of the paper as shown by the responses to each comment. Our responses are mentioned below under each comment raised by the reviewer and it is written in (Times New Roman font, Italic, blue color). Besides, all the updated parts in the manuscript were highlighted in yellow color in order to be easily tracked by the reviewers. | | |
| --- | --- | --- |
| **#** | **Reviewers’ comments** | **Authors’ response** |
| **1** | Thank you for the opportunity to review your work. The paper explores an interesting topic in general, but lacks clarity in its novelty and requires enrichment of the research idea and improvement of its methodology/analytical and theoretical base. | *The aim of this empirical study was to investigate factors that predict and influence the use and acceptance of an EMR system in the healthcare system. We employed an integrated approach using UTAUT and TAM constructs and integrated them with the TAM external factors. This further revealed a significant effect instilled by UTAUT and TAM factors towards BI regarding the use of an EMR system capable of influencing the adoption with four of the external factors proving critical in making the process a success.*  *In terms of* *methodology/analytical and theoretical base, this work, unlike previous empirical investigations that relied solely on SEM analysis, employs a hybrid SEM-ANN strategy based on deep learning to contribute to the literature in general and the healthcare domain in particular. The ANN model outperforms the PLS-SEM model in terms of predictive capability. We infer that ANN analyses' increased predictive power is due to the deep ANN architecture's ability to uncover non-linear correlations between the elements in the theoretical model.* |
| **2** | In the abstract the author(s) wrote “SEM-PLS”, you probably mean PLS-SEM which stands for partial least squares structural equation modeling. Please address this issue where necessary. | *Based on comments from one the reviewers, we defined “PLS-SEM” abbreviation.* |
| **3** | There is a great need to highlight the aim, gap, importance and contributions of this study in the intro section. Based on my readings, the section appears as pile up information without clear bearings. Please try to develop the section to enhance clarity. | *The aim, gap, importance and contributions have been highlighted in introduction section.* |
| **4** | In page 13, line 337. The authors stated that they will use PLS-SEM and ANN. But failed to provide detailed explanation about the pros and cons of the two methods. Below are two valuable studies that you can find details about the usage of the methods. Read and integrate the information available in the papers below:   Abubakar, A. M., Behravesh, E., Rezapouraghdam, H., & Yildiz, S. B. (2019). Applying artificial intelligence technique to predict knowledge hiding behavior. International Journal of Information Management, 49, 45-57.  Yakubu, M. N., Dasuki, S. I., Abubakar, A. M., & Kah, M. M. (2020). Determinants of learning management systems adoption in Nigeria: A hybrid SEM and artificial neural network approach. Education and Information Technologies, 25(5), 3515-3539. | *Thank you for pointing out this comment to us. We provided more details explanations of the pros and cons of the two methods in new section 4.1. The suggested references have been included.* |
| **5** | Information about the language of study, back-translation and pilot survey were not provided by the authors. The left the reviewer wondering, I suggest adding such information with reference and evidences. | *Thank you for bringing these comments to us. In the revised version of the paper we have added the pilot study.* |
| **6** | The authors said they used a cross-sectional and self-rated surveys but did not gauge for common method bias. Why? This has to be done. Also, the authors did not control for non-response bias. Why? | *CMB was gauged (point 5.3.1, Line 527). To test for CMB, Harman's one-factor test was applied. The outcomes show that complete scatterings are 38.63%, which fulfills the guideline of the half. This outcome shows that the current data are liberated from critical CMB issue.*  *“Control for non-response” (Line 336), one paragraph was added to the revised draft to explained how authors deal with this issue. Ex: healthcare facilities visits were directed to ensure the researchers and affiliates of hospitals and clinics administration were properly accustomed and reduce or mitigate the risk of non-respondent bias. At that time, the researchers explained the aims of the research and it’s subject to these staff members.* |
| **7** | I commend the authors approach to reliability, convergent and discriminant validity. Well done. However, the study has several predictors which makes the model vulnerable to multi-collinearity issue. Please consider a test to rule out this problem. | *The constructs have been assessed in terms of multi-collinearity. The variance inflation factor (VIF) of the predictive variables was examined to see whether there was any difficulty with linearity.* |
| **8** | The study reported the beta, t-value, p-value and R-squared, all of which I commend. However, the f-squared values were withheld. Any reason for this, reporting the values will be great. | *The most common measure used to analyze the structural model is the coefficient of determination, so-called R^2^* (1)*. The predictive accuracy of the model is determined using this measure which is computed as the squared correlation between a particular endogenous construct’s actual and predicted values* (2,3)*. In addition, it also signifies the degree of variance in the endogenous constructs validated by every exogenous construct related to it. According to the recommendations provided by* (4)*, when the R^2^ value is more than 0.67, it is perceived as high, whereas the values between 0.33 and 0.67 are considered as moderate, and the values between 0.19 and 0.33 are considered as weak.*  *F-Square is the change in R-Square when an exogenous variable is removed from the model. f-square is effect size (>=0.02 is small; >= 0.15 is medium;>= 0.35 is large)* (5)*.* *Most studies are satisfied with the value of R-squared* *without mentioning the details of the value of the F-Square.* |
| **9** | ANN analyses are well conducted, except my previous comments about the pros and cons, which you can find in the suggested paper. | *The authors are really very grateful to the reviewer for notifying them about these issues. In the revised version of the manuscript, more details on the pros and cons of ANN analyses.* |
| **10** | The implication is too abstract, I am unable to follow nor understand the theoretical or practical implications. Please develop a sub-section and their respective contents as follows: -Theoretical implications -Practical/Managerial implications -Limitations and future research directions | *We are in agreement with the reviewer that the implication section needs to be significantly improved in terms of structure. In the revised version of the paper, the following sub-sections have been added: -Theoretical implications -Practical/Managerial implications -Limitations and future research directions* |

| **Authors’ Response to Reviewer 3:**  The authors are really very grateful to the feedback and comments raised by the reviewer which really assist them to significantly enhance this work and its presentation. The productive and valuable remarks enable us to update many parts of the paper as shown by the responses to each comment. Our responses are mentioned below under each comment raised by the reviewer and it is written in (Times New Roman font, Italic, blue color). Besides, all the updated parts in the manuscript were highlighted in yellow color in order to be easily tracked by the reviewers. | | |
| --- | --- | --- |
| **#** | **Reviewers’ comments** | **Authors’ response** |
| **1** | What is the research question? | *The research questions have been included at the end of the introduction section.*  *“Therefore, our research seeks to answer the following research questions:*   - *What are the factors that affect the EMR adoption in healthcare?* - *How the most widely used external factors of TAM & UTAUT are affect the EMR adoption in healthcare?* |
| **2** | The results didn't provide the ranking of the factors in terms of their significance, See the following references which is also missing in the paper.  Sohaib, O., Hussain, W., Asif, M., Ahmad, M. and Mazzara, M., 2019. A PLS-SEM neural network approach for understanding cryptocurrency adoption. IEEE Access, 8, pp.13138-13150.  Alharbi, A., & Sohaib, O. (2021). Technology readiness and cryptocurrency adoption: PLS-SEM and deep learning neural network analysis. IEEE Access, 9, 21388-21394. | *The authors are really very grateful to the reviewer for notifying them about these issues. In the revised version of the manuscript, section 5.3.4 Importance-Performance Map Analysis provides the ranking of the factors in terms of their significance.*  *Importance-Performance Map Analysis is an improved technique used to analyse the results of the PLS-SEM approach. IPMA involves the use of a target variable for the analysis, which in this case is the actual use of an EMR system. Figure 7 shows the IPMA results for the study’s factors influencing the use of EMR. According to the analysis, the behavioural intention has the highest score in both performance and importance measures, while self-efficacy and performance expectancy has the lowest scores in performance measures and importance measures respectively.*  *The suggested references have been included.* |

**References**

1.	Dreheeb AE, Basir N, Fabil N. Impact of System Quality on Users’ Satisfaction in Continuation of the Use of e-Learning System. Int J e-Education, e-Business, e-Management e-Learning. 2016;6(1):13.

2.	Hair Jr JF, Hult GTM, Ringle C, Sarstedt M. A primer on partial least squares structural equation modeling (PLS-SEM). Sage Publications; 2016.

3.	Senapathi M, Srinivasan A. An empirical investigation of the factors affecting agile usage. In: Proceedings of the 18th international conference on evaluation and assessment in software engineering. ACM; 2014. p. 10.

4.	Chin WW. The partial least squares approach to structural equation modeling. Mod methods Bus Res. 1998;295(2):295–336.

5.	Cohen J. Statistical power analysis for the behavioral sciences . Hilsdale. NJ Lawrence Earlbaum Assoc. 1988;2.
