# Supplementary material for: Determinants predicting the electronic medical record adoption in healthcare: A SEM-Artificial Neural Network approach
Source: PLoS One. 2022 Aug 16;17(8):e0272735. doi: 10.1371/journal.pone.0272735 (PMC9380954; doi:10.1371/journal.pone.0272735)
Supplement: Attachment — Submitted filename: Response to reviewers.docx [file pone.0272735.s002.docx]

**Original Manuscript ID:** PONE-D-22-05312

**Original Article Title:** “Determinants Predicting the Electronic Medical Record Adoption in Healthcare: A SEM- Artificial Neural Network Approach”

**To:** PLOS ONE Editor

**Re:** Response to reviewers

Dear Editor,

The authors are really very grateful to the Journal Editor efforts in essentially discussing the paper reviews with the reviewers and identifying the further significant points for enhancements. The authors would also like to sincerely thank the reviewers for their valuable remarks and careful feedback which helped them to significantly enhance this work and its presentation. Regardless of the final outcome, the authors sincerely thank the editor and reviewers for supporting them. The productive and valuable remarks enable them to update many parts of the paper as shown by the responses to each comment. Besides, all the updated parts in the manuscript were highlighted in yellow color in order to be easily tracked by the editor and reviewers.

Thank you again for allowing a resubmission of our manuscript, with an opportunity to address the reviewers’ comments.

We are uploading (a) our point-by-point response to the comments (below) (response to reviewers), (b) an updated manuscript with yellow highlighting indicating changes (*Revised Manuscript with Track Changes*), and (c) a clean updated manuscript without highlights (*Manuscript).*

Best regards,

Almarzouqi et al.

| **Authors’ Response to Reviewer 3:**  The authors are really very grateful to the feedback and comments raised by the reviewer which really assist them to significantly enhance this work and its presentation. The productive and valuable remarks enable us to update many parts of the paper as shown by the responses to each comment. Our responses are mentioned below under each comment raised by the reviewer and it is written in (Times New Roman font, Italic, blue color). Besides, all the updated parts in the manuscript were highlighted in yellow color in order to be easily tracked by the reviewers. | | |
| --- | --- | --- |
| **#** | **Reviewers’ comments** | **Authors’ response** |
| **1** | The authors mentioned in the response that the suggested references are included for IPMA. But, I didn't find the references as it's not included.  Sohaib, O., Hussain, W., Asif, M., Ahmad, M. and Mazzara, M., 2019. A PLS-SEM neural network approach for understanding cryptocurrency adoption. IEEE Access, 8, pp.13138-13150.  Alharbi, A., & Sohaib, O. (2021). Technology readiness and cryptocurrency adoption: PLS-SEM and deep learning neural network analysis. IEEE Access, 9, 21388-21394. | *The suggested references have been included.* |
